# Place de la radiothérapie dans les séminomes testiculaires de stade I: à propos de 25 cas

**DOI:** 10.11604/pamj.2016.25.53.7586

**Published:** 2016-09-30

**Authors:** Abdellah Aissa, Elamin Marnouche, Hanan Elkacemi, Tayeb Kebdani, Noureddine Benjaafar

**Affiliations:** 1 Service de Radiothérapie, Institut National d’Oncologie, Université Mohamed V Rabat, Maroc

**Keywords:** Séminome testiculaire stade I, radiothérapie adjuvante, irradiation lombo-aortique, chimiothérapie adjuvante, surveillance, Stage I testicular seminoma, adjuvant radiotherapy, lombo-aortic irradiation, adjuvant chemotherapy, monitoring

## Abstract

Notre travail se proposait de rapporter les résultats d’une étude rétrospective, descriptive, portant sur 25 séminomes testiculaires de stade I et de préciser la place de la radiothérapie dans la prise en charge de cette entité. Entre janvier 2001 et décembre 2009, 25 patients atteints d'un séminome testiculaire de stade I ont été pris en charge au service de radiothérapie de l’institut national d’oncologie de Rabat. L’orchidectomie première a été réalisée par voie inguinale. Le bilan d’extension initial comportait un dosage de bHCG totale, d’alphafoetoprotéine, et une exploration des aires ganglionnaires sus- et sous-diaphragmatiques par une tomodensitométrie. L’irradiation adjuvante a été délivrée au moyen d’un accélérateur linéaire. L'âge médian est de 33 ans (18-52 ans). La tumeur testiculaire siégeait à droite chez 16 malades et à gauche chez les 9 autres. La radiothérapie était délivrée dans les aires ganglionnaires lomboaortiques pour 18 patients, lomboaortiques et iliaques homolatérales pour les 7 autres et ceci par deux faisceaux antéropostérieurs, délivrant une dose de 20 à 25 Gy en 10 à 14 fractions. La tolérance immédiate était excellente. La durée médiane de surveillance était de 73 mois. Vingt trois patients sont actuellement vivants, en situation de rémission complète. Un patient a rechuté au niveau pulmonaire 22 mois après la fin de sa radiothérapie. Un patient a été perdu de vue. Il n’a pas été observé de toxicité à long terme, en particulier gastro-intestinale. Aucune tumeur ou pathologie hématologique secondaire n’a été rapportée. La radiothérapie prophylactique reste le traitement adjuvant de référence des séminomes de stade I. La tolérance immédiate est satisfaisante et l’augmentation du risque de cancer secondaire est négligeable par rapport au bénéfice thérapeutique. Toutefois une surveillance armée ainsi qu’une chimiothérapie adjuvante avec un cycle de carboplatine sont aussi efficaces.

## Abstract (en)

We conducted a retrospective, descriptive study of 25 stage I testicular seminomas to clarify the role of radiotherapy in the management of this disease. Between January 2001 and December 2009, 25 patients with stage I testicular seminoma were treated in the Radiotherapy Department at the National Institute of Oncology in Rabat. Primary orchidectomy was performed via the inguinal route. Initial staging was based on total beta-hCG dosage, alpha-fetoprotein dosage and exploration of superior diaphragmatic and sub-diaphragmatic lymph nodes using tomodensitometry. Adjuvant radiotherapy was delivered using linear accelerator. The median age was 33 years (18-52 years). Testicular tumor involved the right side in 16 patients and the left side in 9 patients. Radiotherapy was delivered to lombo-aortic lymph nodes in 18 patients, lombo-aortic and ipsilateral iliac lymph nodes in 7 patients, using 2 anterior-posterior beams, with delivery of 20-25 Gy in 10-14 fractions. Immediate tolerance was excellent. The average monitoring period was 73 months. Twenty three patients are currently alive in complete remission. One patient developed a pulmonary relapse 22 months after the end of the radiotherapy. One patient was lost to follow-up. Long-term toxicity, especially gastrointestinal toxicity, was not observed. No tumor or secondary hematologic disease was reported. Prophylactic radiotherapy remains the standard adjuvant treatment of stage I seminomas. Immediate tolerance is satisfactory and an increased risk for secondary cancer is negligible compared to the therapeutic benefit. However, strict monitoring and one cycle of carboplatin-based adjuvant chemotherapy are also effective.

## Introduction

Les séminomes représentent environ 40 % des tumeurs germinales du testicule. La radiothérapie est l’une des options thérapeutiques en situation adjuvante après orchidectomie par voie inguinale, dans les séminomes testiculaires de stade I. Nous rapportons dans ce travail l’expérience du service de radiothérapie de l’institut national d’oncologie de Rabat.

## Méthodes

Cette étude a porté sur une série rétrospective de 25 patients atteints d'un séminome testiculaire de stade I pris en charge au service de radiothérapie de l’institut national d’oncologie de Rabat de janvier 2001 à décembre 2009. Les différentes données diagnostiques et thérapeutiques ont été recueillies des dossiers cliniques, du système de vérification et d’enregistrement Mosaiq et du logiciel de planification de traitement pour radiothérapie XiO. La stratégie thérapeutique adoptée par le service est calquée sur les guidelines internationaux. L’orchidectomie première a été réalisée par voie inguinale. Le bilan d’extension initial comportait un dosage de bHCG totale, d’alphafoetoprotéine, et une exploration des aires ganglionnaires sus- et sous-diaphragmatiques par une tomodensitométrie. L’irradiation adjuvante a été délivrée au moyen d’un accélérateur linéaire. Le premier temps de la préparation d’un plan de traitement pour une radiothérapie était le positionnement du malade en décubitus dorsal, bras le long du corps avec des moyens de contention simple. Un plan de référence était défini sur le patient par trois points (un antérieur et deux latéraux) qui sont tatoués. Un scanner était ensuite réalisé, en s’assurant que le plan de référence passe exactement par une des coupes scanner. Les images scanographiques étaient transférées vers un système de planification de traitement pour radiothérapie. Le volume cible incluait la chaîne ganglionnaire lomboaortique tracée sur chaque coupe chez 15 patients. Ce volume a été associé à une irradiation iliaque homolatérale à la tumeur chez les 10 autres patients. Selon la période de traitement, la dose était de 20 Grays en fractionnement classique chez 16 patients et de 25,2 Grays en 14 fractions de 1,8 Grays chez 9 patients. L’irradiation a été délivrée au moyen des photons X de haute énergie, par deux faisceaux antéropostérieurs. Une consultation hebdomadaire a été instituée pendant toute la durée de l’irradiation, permettant l’appréciation clinique de la toxicité immédiate. La surveillance au long cours a été effectuée par le radiothérapeute et l’urologue, en alternance.

## Résultats

L’âge médian était de 33 ans (18-52 ans). Des antécédents testiculaires ont été identifiés chez quatre patients (trois cas de cryptorchidie et un cas d’atrophie testiculaire). La tumeur testiculaire siégeait à droite chez 16 malades (64% des cas) et à gauche chez les 9 autres (26%). La radiothérapie était bien tolérée et tous les malades ont terminé leur traitement. La toxicité immédiate a été essentiellement digestive, à type de nausées et vomissements jugulé par un traitement symptomatique. Avec un recul médian de 73 mois (60-114), vingt-trois patients sont actuellement en situation de rémission complète, un patient a rechuté au niveau pulmonaire 22 mois après la fin de sa radiothérapie et un patient a été perdu de vu après un suivie de 38 mois sans aucun événement noté. Il n’a pas été observé de toxicité à long terme, en particulier gastro-intestinale. Aucune tumeur ou pathologie hématologique secondaire n’a été rapportée. Le Tableau 1 résume les différentes caractéristiques de la population et les résultats de ce travail.

**Tableau 1:** Caractéristiques de la population et résultats (n=25)

Caractéristique	Valeur
Age (années)	33(18-52)
Antécédents	
Cryptorchidie	3(12%)
Atrophie testiculaire	1(4%)
Localisation	
Testicule droit	16(64%)
Testicule gauche	9(36%)
Irradiation lomboaortique et pelvienne	7(28%)
Irradiation lomboaortique seule	18(72%)
Dose	
20 Gy/ 10 fractions	16(64%)
25,2 Gy/ 14 fractions	9(26%)
Suivi (mois)	63(60-114)
Taux de rechute	4%

## Discussion

La prise en charge thérapeutique des séminomes de stade I soulève encore aujourd’hui de nombreuses questions [1]. Les recommandations thérapeutiques proposent la réalisation d’une surveillance, d’une chimiothérapie ou d’une radiothérapie adjuvante. Ces différentes options thérapeutiques ne sont pas équivalentes concernant la survenue d’effets secondaires et le risque de récidive [2]. Le traitement standard adjuvant a longtemps consisté en une irradiation adjuvante des aires ganglionnaires de drainage impliquées ou à risque de dissémination micro-métastatique [3]. Après l'abandon du champ d'irradiation médiastinal et jusque dans les années 1990, les champs d'irradiation standard comprenaient les aires ganglionnaires iliaques homolatérales, obturatrices, les aires ganglionnaires lomboaortiques sans irradier les aires ganglionnaires inguinales (à partir des années 1980). La limite supérieure du champ lomboaortique était D10-D11. Latéralement le champ d'irradiation couvrait les hiles rénaux avec un cache rénal mis en place après 18 à 20 Gy délivrés [4]. Cette irradiation permettait d’obtenir un taux de survie sans rechute de 96% et un taux de survie globale de 98% avec cependant une toxicité aiguë contraignante et un risque non négligeable de cancer radioinduit à long terme [3, 5]. Actuellement, les modalités de la radiothérapie ont fortement évolué. Le standard thérapeutique est aujourd’hui une irradiation lombo-aortique conformationnelle de 20 Gy en 10 séances et a une efficacité comparable aux protocoles antérieurs [6, 7]. Ainsi, les essais T10, T11 du Medical Council ont démontré que le champ d'irradiation doit se limiter aux aires ganglionnaires lomboaortiques avec un taux de survie sans récidive comparables à ceux obtenus avec une irradiation en dog-leg classique [8]. Dans notre série, Le volume cible, chez les malades inclut âpres ces résultats, comprenait uniquement la chaine ganglionnaire lomboaortique. Concernant la dose prescrite, l’ancien standard était une irradiation à la dose de 30 Gy en 15 fractions de 2 Gy sur 3 semaines [9, 10]. Une désescalade de dose a été approuvée par l’essai du Medical Research Council (MRC) TE18 et de l’European Organisation for the Research and Treatment of Cancer Trial (EORTC) 30942, qui comparait pour des volumes semblables une dose de 30 Gy en 15 fractions sur trois semaines à une dose de 20 Gy en dix fractions sur deux semaines. L’efficacité en termes de rechute était la même avec un suivi de quatre ans. La toxicité aigue était réduite dans le bras 20 Gy en dix fractions [3, 7]. Dans notre série, selon la période de traitement, la dose était de 20 Grays en fractionnement classique chez 16 patients et de 25,2 Grays en 14 fraction de 1,8 Grays chez 9 patients. Cette évolution de la radiothérapie a permis de limiter les toxicités. Les principales toxicités aiguës retrouvées après radiothérapie sont digestives (nausées, vomissements, diarrhées, gastrites) et les toxicités tardives « historiques » ont quasiment disparu [2, 11]. Cependant, l’hypofertilté secondaire à la radiothérapie lombo-aortique atteint toujours 11 % et le risque de second cancer n’est pas négligeable [12]. Ce risque serait majoré chez les patients jeunes et après chimiothérapie [13, 14]. Vu ces effets tardifs de la radiothérapie et vu la chimiosensibilité constatée dans les séminomes avancés [2, 15, 16], la chimiothérapie a été reconsidérée dans le traitement des séminomes testiculaires de stade I. Ceci a été confirmé par l’étude randomisée MRC TE19/ EORTC 30982 qui comparait une irradiation lombo-aortique de 20-30 Gy à un cycle de carboplatine (AUC 7) pour 1477 patients présentant un séminome de stade I. Le taux de rechute est quasiment identique dans les 2 bras (5% et 4%). le nombre de nouveaux cancers primitifs a été de 1% dans le bras Carboplatine et de 3% dans le bras radiothérapie [17]. Les séries de patients surveillés après orchidectomie montrent qu'un traitement adjuvant peut être évité chez 80% des patients. Ce choix permet d’éviter tous les effets secondaires à court et à long terme que l’on peut attribuer aux autres options thérapeutiques, qu’il s’agisse de la radiothérapie ou de la chimiothérapie [2].

## Conclusion

La radiothérapie prophylactique reste le traitement adjuvant de référence des séminomes de stade I. La tolérance immédiate est satisfaisante et l’augmentation du risque de cancer secondaire est négligeable par rapport au bénéfice thérapeutique. Toutefois une surveillance armée ainsi qu’une chimiothérapie adjuvante avec un cycle de carboplatine sont aussi efficaces. L’intérêt donc, d’une information précise au patient des différentes modalités thérapeutiques, de leurs avantages et de leurs inconvénients car ce sont principalement ces exigences qui guideront le choix entre surveillance, radiothérapie et chimiothérapie adjuvantes.

### Etat des connaissances actuelles sur le sujet

- La prise en charge thérapeutique des séminomes de stade I soulève encore aujourd’hui de nombreuses questions;
- Les recommandations thérapeutiques proposent la réalisation d’une surveillance, d’une chimiothérapie ou d’une radiothérapie adjuvante.

### Contribution de notre étude à la connaissance

- La radiothérapie prophylactique reste le traitement adjuvant de référence des séminomes de stade I;
- Une surveillance armée ainsi qu’une chimiothérapie adjuvante avec un cycle de carboplatine sont aussi efficaces;
- Une information précise au patient des différentes modalités thérapeutiques, de leurs avantages et de leurs inconvénients.
